# A Self-Powered Flexible Thermoelectric Sensor and Its Application on the Basis of the Hollow PEDOT:PSS Fiber

**DOI:** 10.3390/polym12030553

**Published:** 2020-03-03

**Authors:** Limin Ruan, Yanjie Zhao, Zihao Chen, Wei Zeng, Siliang Wang, Dong Liang, Jinling Zhao

**Affiliations:** 1 National Engineering Research Center for Analysis and Application of Agro-Ecological Big Data, Anhui University, No. 111 Jiulong Road, Hefei 230601, China; 18803838423@163.com (Y.Z.); youfmail@163.com (W.Z.); sliang_wang@163.com (S.W.); 2 School of Electronics and Information Engineering, Anhui University, No. 111 Jiulong Road, Hefei 230601, China; Chen2130418@163.com

**Keywords:** thermoelectric (TE) fiber, PEDOT:PSS, sensor, self-powered, flexibility

## Abstract

The thermoelectric (TE) fiber, based on poly(3,4-ethylenedioxythiophene):poly(styrenesulfonate) (PEDOT:PSS), which possesses good flexibility, a low cost, good environmental stability and non-toxicity, has attracted more attention due to its promising applications in energy harvesting. This study presents a self-powered flexible sensor based on the TE properties of the hollow PEDOT:PSS fiber. The hollow structure of the fiber was synthesized using traditional wet spinning. The sensor was applied to an application for finger touch, and showed both long-term stability and good reliability towards external force. The sensor had a high scalability and was simple to develop. When figures touched the sensors, a temperature difference of 6 °C was formed between the figure and the outside environment. The summit output voltages of the sensors with 1 to 5 legs gradually increased from 90.8 μV to 404 μV. The time needed for the output voltage to reach 90% of its peak value is only 2.7 s. Five sensors of legs ranging from 1 to 5 were used to assemble the selector. This study may provide a new proposal to produce a self-powered, long-term and stable skin sensor, which is suitable for wearable devices in personal electronic fields.

## 1. Introduction

Thermoelectric (TE) materials can directly convert heat energy into electrical energy. They generate electricity through the dissipated heat in the environment and allow resources to be fully utilized. The energy conversion efficiency of TE materials is determined by a dimensionless figure of merit (*ZT*) derived from Equation (1). In addition, the power factor (PF) expressed as *S*^2^*σ* can be used to characterize the performance of TE materials. Compared with inorganic TE materials, organic TE materials have attracted considerable interest recently due to their unique properties, such as flexibility, a low cost, low thermal conductivity, good environmental stability and non-toxicity [1,2,3]. In particular, great efforts have been made on the design of flexible electric materials and new structural constructions to achieve flexibility. Conductive polymers including polyacetylene, polypyrrole, polyaniline and poly(3,4-ethylenedioxythiophene) (PEDOT) derivatives possess unique advantageous properties such as high conductivity [4,5] and compatibility with wearable applications [6,7].

(1) $$ZT=S^{2}\sigma T/k$$

where *S* is the Seebeck coefficient, *σ* is electrical conductivity, *T* is absolute temperature and *k* is thermal conductivity.

Compared with other polymeric TE materials, PEDOT:poly(styrenesulfonate) (PEDOT:PSS) has an outstanding water solubility, a potential to achieve good electrical conductivity [2,8] and a tunable Seebeck value. Thus, they have been successfully applied to TE devices [9,10,11]. As an organic-based TE material, PEDOT:PSS is suitable for the preparation of thin-film or fiber devices through chemical modification and complex materials [12,13]. The Seebeck value of the PEDOT:PSS film is reported to be 19.4 μV/K [14]. A recent study [15] shows that a remarkable increase of a Seebeck value can be reached of –18.2 mV/K by using a copper (II) chloride (CuCl_2_) doping. In comparison with the low thermal conductivity of a only 0.4 W/mK [16] and good electrical conductivity (4380 s/cm) for film [17], PEDOT:PSS has become one of the most promising candidates for TE materials. After several years of development, great achievements have been made in developing functional materials by treating the materials for energy harvesting with different solvents [18,19] and the fabrication of optical TE generators with high output voltages [10,19] This is a great challenge due to the strong demand for high flexibility and good TE properties, but also shows promising prospects for the development of wearable modules for utilizing energy. 

The TE fiber has aroused great interest in exploring potential applications for wearable technologies. Compared with film or bulk materials, TE fibers with a small physical size arranged from several to hundred microns can enable various devices to be smaller and become more lighter and more portable. They can also possess high flexibility and favorable electrical properties. Recently, a reported ~15 micron PEDOT:PSS fiber [17] possessed higher flexibility with an electrical conductivity of 3828 S/cm, which is compatible with the value of an ultra-thin PEDOT:PSS film. Different kinds of TE fibers have emerged, and considerable attention has been given to SWCNT/PVDF composite fibers [14,16], CNT-GO fibers [20], and glass-semiconductor fibers [21] for applications in energy harvesting, thermal sensing, and positioning. With years of development, major achievements have been obtained for flexible sensors towards promising applications in personal health [22,23], artificial intelligence systems [24,25,26], wearable motion detection [27,28], and wearable healthcare devices [29]. The essential characteristics for next-generation flexible sensors, such as a high resolution, ultrasensitivity, and a long-life cycle, have drawn considerable attention in future human-oriented technologies. This study focused on an exploration of a figure touching a sensor based on PEDOT:PSS fibers. Here, a flexible self-powered skin sensor is presented, which possesses high reliability, reasonable stability, and good mechanical properties. These sensors with different fiber leg numbers can output a voltage based on temperature differences. In general, the skin of a human being maintains a certain temperature of around 33 °C. When the figure touches the sensors, a temperature difference of 6 °C rapidly develops. Owing to the TE properties of PEDOT:PSS fibers, the voltage can be generated rapidly once one touches the sensor. These TE fibers prepared by using wet spinning were encapsulated on a low-thermal-conductivity polydimethylsiloxane (PDMS) layer, which acted as a heat barrier. When touching one side of the sensor with a finger, a highly selective response could be obtained by providing a discriminant output voltage between human skin and the environment. Our study may provide a reference for the development of a self-powered, long-term, and stable skin sensor for wearable devices in personal electronic fields.

## 2. Experimental

### 2.1. Materials and Reagents

PEDOT:PSS aqueous solution (Clevios PH1000) was purchased from Shanghai JingNian Chemical Co., Ltd. (Shanghai, China). Sulfuric acid (H_2_SO_4_, 98%) and ethanol (EtOH, 99.7%) were analytical grade reagents obtained from Sinopharm Chemical Reagent Co., Ltd. (Shanghai, China). Ethylene glycol (EG, 98%) was analytical grade and was purchased from Shanghai Macklin Biochemical Co., Ltd. (Shanghai, China). PDMS (184-silicone elastomer) was purchased from Dow Corning Co., Ltd. (Midland, MI, US). All chemicals were used without further purification.

### 2.2. Preparation of PEDOT:PSS Fber and PDMS Layer

Wet spinning of PEDOT:PSS fibers: four types of syringe needles (denoted as N1, N2, N3, and N4) were used in the study. Their inner and outer diameters were 0.51 and 0.81 mm, 0.90 and 1.26 mm, 1.45 and 1.81 mm, and 2.40 and 2.75 mm, respectively. The spinning speed was set at 0.01, 0.10, 0.20, and 0.35 mL/min. To increase the viscosity of ink, 8 mL of the PEDOT:PSS aqueous solution was evaporated at 50 °C in vacuum for 8, 12, and 16 h. The spinning formulation was loaded into a 10 mL syringe and spun into a H_2_SO_4_ coagulation bath (the volume ratio for H_2_SO_4_ and water was 1:2) though a metal needle. The flow rate of the ink was controlled by using a syringe pump. For the fibers: (1) with EG post-treatment, the obtained fibers from syringe pump were stirred in a H_2_SO_4_ coagulation bath for 10 min, and washed with a mixed solution of H_2_O/ethanol. After EG post-treatment, the fibers were hung up with a copper wire (the diameter is 2 mm) and dried in a drying box at 70 °C; (2) without EG post-treatment for comparative analysis, the obtained fibers from syringe pump were stirred in a H_2_SO_4_ coagulation bath for 10 min, and washed with a mixed solution of H_2_O/ethanol. The drying procedure was similar to that with EG post-treatment. The fibers were hung up with a copper wire and dried in a drying box at 70 °C. Ten minutes later, fibers were all dried. The edge attached to the copper wire was cut and the rest part of fiber was used to perform the next analyses.

PDMS layer: the PDMS was prepared by curing a mixed PDMS prepolymer with coagulant in a 10:1 mass ratio. A PDMS prepolymer was prepared, and 1 g of coagulant was mixed into 10 g of PDMS dispersion with a magnetic stirrer for 2 h. The mixed solution was then placed in a petri dish and allowed to stand overnight at 5 °C to remove air bubbles. The mixed solution was stored at room temperature for 24 h. Finally, the PDMS layer was carefully peeled off the petri dish. Here, the PDMS layer was used as the insulating layer and heat barrier of the hot–cold side of the sensor.

### 2.3. Preparation of PEDOT:PSS Sensors and Selector

Sensors: legs 1 to 5 of PEDOT:PSS fibers were twined on two sides of the PDMS layer. The fibers were connected with copper wires in series, and the aligned contact points between the fiber and the copper wire were fixed with silver glue to decrease the contact resistance. Then, the sensors were encapsulated by polyimide (PI) high temperature adhesive tape.

Selector: The prepared sensors were connected in parallel and assembled on a flexible printed circuit board. Then, a series of connected switches was placed on the bottom of the sensors. Finally, the sensors were encapsulated with PI film on the PDMS layer.

### 2.4. Characterization

The morphologies and microstructures were characterized by using scanning electron microscopy (SEM, Hitachi S-4800, Tokyo, Japan). The surface chemical states and properties of energy levels were analyzed by using X-ray photoelectron spectroscopy of (XPS, Thermo-Fisher Scientific ESCALAB 250Xi, Waltham, MA, US). The sample was heated using a self-made temperature control device, and the output TE voltage was measured with a KEYSIGHT 34470A nanovoltmeter (KEYSIGHT Technologies, Santa Rosa, CA, US) using the gold-plated brass probe. The accuracy of the temperature control device and thermal insulation of the MS layer were verified using a TESTO 865 thermal imager (TESTO AG, Schwarzwald, Germany). The TE properties of the sensors and selector were determined through the temperature difference (6 °C) between the finger and the outside environment (27 °C).

## 3. Results and Discussion

### 3.1. Morphological and Structural Characterizations of PEDOT:PSS Fiber

**Figure 1:**
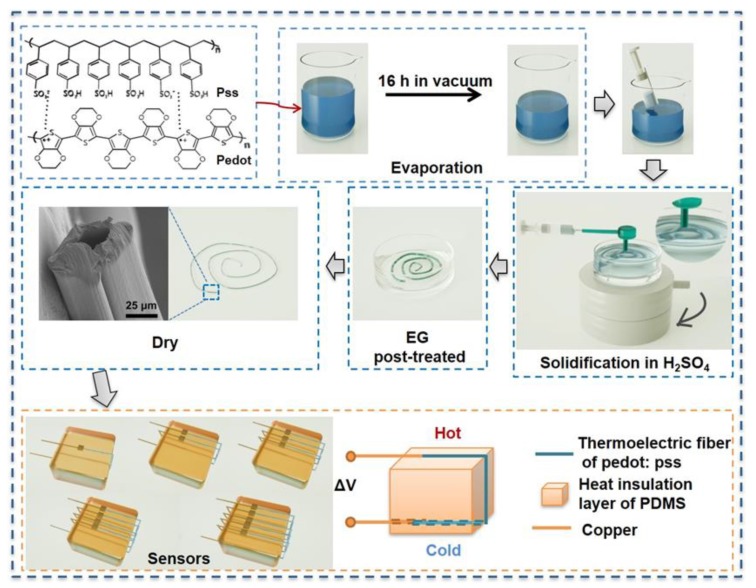
Fabrication scheme for producing the thermoelectric (TE) fiber sensor.

The whole fabrication scheme for the TE fiber sensor is shown in Figure 1. The PEDOT:PSS fiber was prepared via the wet-spinning method. Previous studies [30,31] have suggested that improved electrical properties can be obtained by removing PSS through post-treatment of organic polar solvents. PSS as an organic linker is an insulator and has a good solubility in EG. Therefore, EG was used to remove the PSS to increase the electrical properties in this study. Here, the PDMS layer acts as the heat barrier. One side with heat injected is the hot side, whereas the other is utilized as the cold side. On the basis of the different quantities of PEDOT:PSS fiber loaded on the PDMS surface, the as-prepared sensors can directly output the voltage at a certain temperature difference. The sensitivity of temperature difference is essential for the primary signal response of a TE fiber sensor.

**Figure 2:**
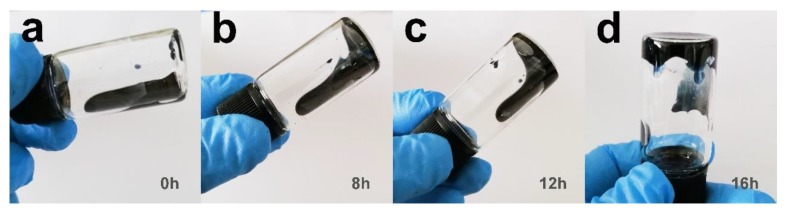
Optical images of PEDOT: PSS solutions condensed with (**a**) 0 h, (**b**) 8 h, (**c)** 12 h, (**d**) 16 h.

**Figure 3:**
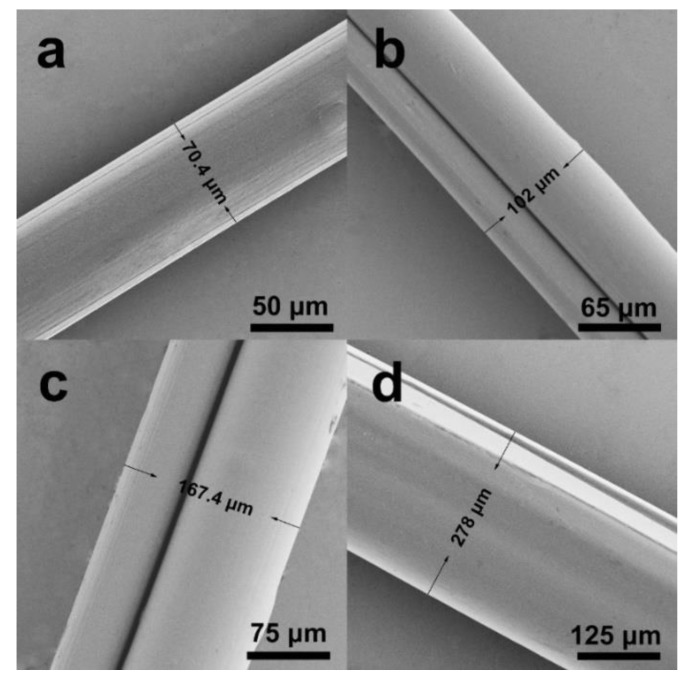
SEM images of PEDOT: PSS fibers of (**a**) f1, (**b**) f2, (**c**) f3 and (**d**) f4 with EG post-treatment.

The condensed time of the PEDOT:PSS solution is the most important parameter for wet spinning because it influences the fiber’s morphology. The time duration in this study ranged from 0 h to 16 h. The optical images of PEDOT:PSS concentration solutions for 0, 8, 12, and 16 h are shown in Figure 2. When the solution was condensed for 8 h, the fibers were easy to fabricate into an uneven shape. By contrast, the solution concentrated over 16 h became much denser and was filled with several tiny bubbles when transferred into the syringe. The bubbles were difficult to remove during wet spinning, which influenced the fiber’s electrical properties. In this study, the optimal concentration time was 12 h. In addition, the resultant fibers with diameters of 70.4, 102.0, 167.4, and 278.0 μm were denoted as f1, f2, f3, and f4, respectively (Figure 3), which were obtained by using four types of spinning needles (N1, N2, N3, and N4). 

**Figure 4:**
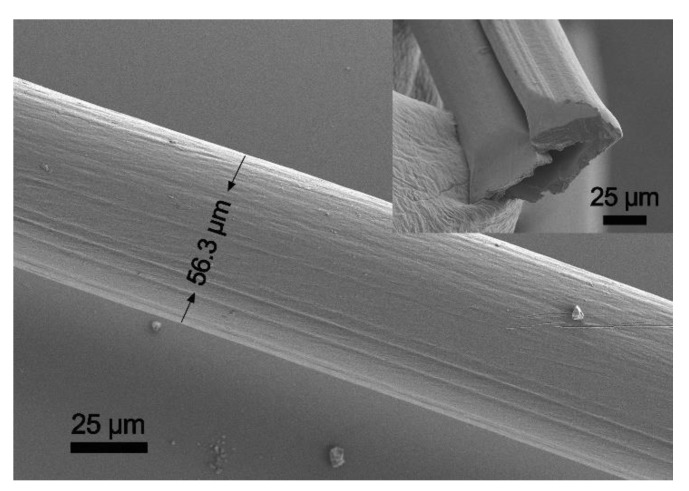
SEM image and its cross-section image of the fiber without EG post-treatment.

**Figure 5:**
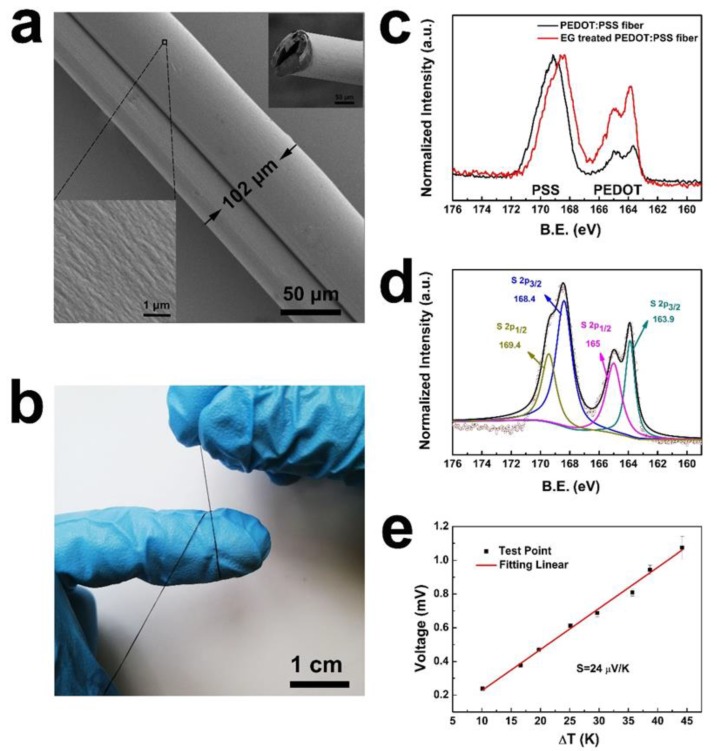
(**a**) SEM image of the PEDOT:PSS fiber. The inserted are the enlarged morphology of fiber surface and cross-section image. (**b**) Optical image of the PEDOT:PSS fiber. (**c**) XPS spectra of S 2p atomic orbitals of PEDOT:PSS before and after EG post-treatment. (**d**) Fitted curve of the S 2p atomic orbital spectrum. (**e**) Relationship diagram with output voltage of the fiber contrasted to the temperature gradient.

A hollow structure can be observed in both fibers without EG post-treatment (Figure 4) and with EG post-treatment (Figure 5a). Based on analysis of a large number of SEM images, the fibers were all hollow. Such a hollow structure may be influenced by the needles, spinning speed, ink concentration, temperature in drying process, and even the dry method. The wet fibers extracted from the syringe pump was prone to stretch before they solidified. Therefore, when the prepared ink of PEDOT:PSS was spun into the coagulation bath, the fibers became more and more flat with the rotation of the H_2_SO_4_ aqueous bath. After EG post-treatment, the fibers were hung up using a copper wire and dried in a drying box at 70 ºC. Ten minutes later, when the fibers were all dried, they were curled and the hollow structure was formed. The hollow fibers might have come from the drying procedure rather than the EG post-treated procedure. Owing to the inner hollow, the surface area was larger than that of the cylindrical one with the same diameter. The inner fiber had also been post-treated by EG. This meant that an increased surface area might be beneficial towards the removement of more insulating PSS.

The SEM image of the PEDOT:PSS fiber with EG post-treatment (f2) is shown in Figure 5a, and the typical texture is observed on the fiber surface. Of note, most of the synthesized fibers were hollow. The section feature and surface structure of the fiber are shown in the inserted picture of Figure 5a. It is a typical hollow fiber with good flexibility (Figure 5b). The resistance of the f2 fiber after EG post-treatment decreased to 54.6 Ω in average for 10 samples at the same length, whereas that of the fiber after H_2_SO_4_ solidification was averaged to be 265.8 Ω for 10 samples. An apparent drop in resistance was observed, indicating that the electrical property of the fiber after EG post-treatment is greatly improved. 

According to a previous report [32], the electrical properties of the PEDOT:PSS fiber can be improved by post-treatment with a polar solvent, which removes excess PSS from the fibers. Generally speaking, PSS in PEDOT:PSS fibers are considered as an insulating component or can cause a conformational change in the twisted PEDOT chains to increase the doping effect. PEDOT:PSS gel particles possess a PEDOT-rich core and shell on the basis of the PEDOT:PSS structure [33,34,35]. The PSS-rich region surrounds the PEDOT-rich region to form domains. Analysis of the high-resolution SEM image of the PEDOT:PSS fiber surface shows that the diameters of the prepared fiber domains are distributed from several micrometers, which are much larger than those of the reported PEDOT:PSS thin film [36]. The highly conductive PEDOT-rich regions interconnect to form carrier transport paths known as percolation paths [14,32,37]. The charge flowing through the PEDOT:PSS fiber must cross the insulating PSS-rich domain boundaries via hopping. Larger domains or better interconnection between the domains facilitate the hopping of the charge carriers [38]. 

The XPS spectra in Figure 5c show the contrast curves of the S 2p atomic orbitals of PEDOT:PSS before and after EG post-treatment, indicating the chemical composition of the prepared PEDOT:PSS fibers. The most obvious difference lies in the integral area of characteristic peaks between 162 eV and 166 eV, while the quantity of EG can influence the relative content of PEDOT to PSS (thiophene/sulfonate) in the corresponding fibers [18,19]. The surface thiophene/sulfonate ratio directly reflects the surface ratio of PEDOT to PSS. Thus, the ratio of thiophene/sulfonate of PEDOT:PSS fiber without EG treatment is 1:2.6, whereas it increases to 1:1.28 for the thiophene/sulfonate of PEDOT:PSS fiber after EG post-treatment. The phenomenon can be confirmed by phase separation between the chains and the removal of additional PSS with the solvent. However, XPS analysis was only used to identify the stoichiometry for the surface fiber; the stoichiometry of the inner fiber could not be identified. According to the fitted curves of the S 2p spectra in Figure 5d, the peaks at 163.9 (S 2p3/2) and 165 eV (S 2p1/2) represent the PEDOT of the fiber, and those at 168.4 (S 2p3/2) and 169.4 eV (S 2p1/2) represent the PSS of the fiber. Obviously, the increased PEDOT-to-PSS ratio is beneficial to carrier transport and can also increase the conductivity of the PEDOT:PSS fiber. A significant drop in resistance of the fibers can be observed, which can be ascribed to their increased PEDOT-to-PSS ratio.

**Figure 6:**
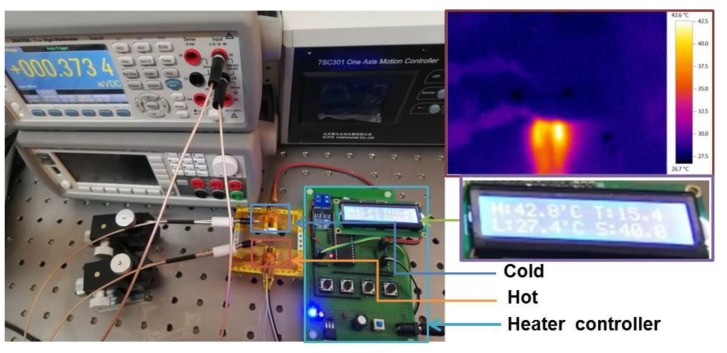
Designed heater with a tunable temperature controller, hot plate, and pair of probe stations.

The relationship curve between the recorded output voltage and temperature gradient is shown in Figure 5e. The red line is the result of the fitted variation trend of output voltage, and it can be confirmed to show a good linear relationship. According to the equation *S*=*ΔV*/*ΔT*, *S* of the tested sample can be estimated, where *ΔV* is the voltage between the hot and cold side while *ΔT* is the temperature difference between the hot and cold sides. The *S* of the PEDOT:PSS fiber was calculated to be approximately 24 μV/K, which is similar to previously reported PEDOT:PSS TE materials [6,10]. Statistics of TE properties of PEDOT related materials were shown in Table 1. The Seebeck value, the physical property coefficient, only relates to the original material rather than the hollow structure of the fiber. Combined with the improved electric properties, we hypothesized that the hollow fiber may have a larger PF than the cylindrical one. To examine the Seebeck coefficient of the prepared fiber, the heater was designed using a tunable temperature controller, hot plate, and detector. Figure 6 shows a digital photo of the designed heater system. The thermal infrared image of the heat source temperature is shown on the right side of Figure 6. The temperature of the hot side was supplied by a ceramic heating element, and the cold side was supported by a ceramic plate. The whole hot plate was insulated and covered with the temperature sensor. The voltage between both sides was detected by a pair of probe stations with the copper-plated gold probe. This heater was applied to generate a temperature difference (*ΔT*) derived from the two sides of the fiber.

### 3.2. TE Fiber Sensors

**Figure 7:**
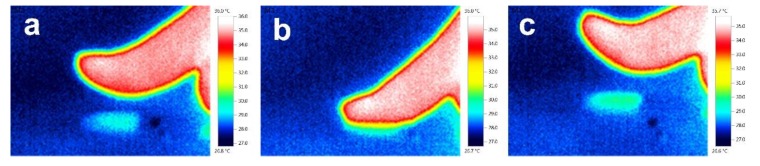
Thermographic images of (**a**) the PMDS layer before touching the PDMS surface, (**b**) the finger pressing onto the surface of the layer for 10 s, and (**c**) the PDMS layer after leaving the surface.

PDMS is a silicon-based organic polymer that behaves as an inert, non-toxic, non-flammable, optically transparent elastomer with low thermal conductivity [39,40]. The thickness, shape, or size of the PDMS layer can be easily controlled. It has been widely used in solid support for sensing applications due to its unique characteristics. Its flexibility can protect the fiber from being damaged to a certain extent, and the thermal insulation properties maintain the temperature difference between both sides of the sensor. Figure 7a shows a thermographic image of the PMDS layer before the finger touches the PDMS surface; Figure 7b shows the image when the finger presses onto the surface of the layer for 10 s; Figure 7c shows the image of the PDMS layer after the finger leaves the surface. As can be seen from the figure, the surface of the PDMS leaves a visible heat mark when the sensor is exposed to the finger at room temperature (27 °C). The PDMS layer provides good thermal insulation, which can isolate the heat transformation from the hot side to the cold side and allows the sensor to maintain a certain temperature difference.

**Figure 8:**
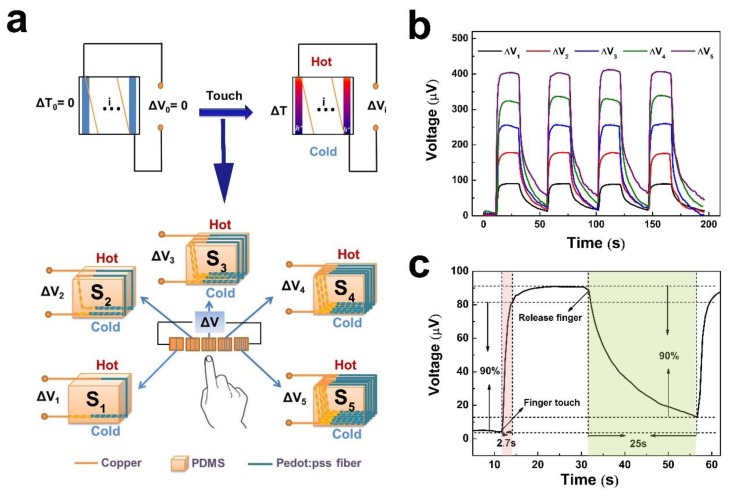
(**a**) Schematic of output voltage measurement of S1, S2, S3, S4, and S5. (**b**) Output voltage of the five sensors compared with time-variant change at the temperature difference of 6 °C between the finger and outside environment. (**c**) Response time of a single TE fiber.

The PEDOT:PSS fiber can be easily bent to assemble on a PDMS layer due to its good flexibility (Figure 5b). The output voltages of the sensors increase as the fiber number increases at the same temperature difference. Therefore, the sensors can be applied to detect five targets on the basis of the response of output voltages. Here, we used five fingers as the testing targets. The five sensors were denoted as S1, S2, S3, S4, and S5. Figure 8a presents the schematic of output voltage measurement of the five sensors. The peak output voltages were represented by ΔV1, ΔV2, ΔV3, ΔV4, and ΔV5. Each sensor was tested for four pulses, and the curves of output voltages compared with time are shown in Figure 8b. Generally, living human skin is generally a permanent heat source maintained at a temperature of 33 °C [41]. Our test temperature of the environment was 27 °C. The values of ΔV1 to ΔV5 increased, which were 90.8, 178.9, 256.8, 336.7, and 404 μV at the temperature difference of 6 °C when the finger touched the sensors. These results suggested that the output voltages exhibited a positive correlation with the fiber number. In addition, no significant deviation in the output voltage for each sensor was observed, which may be due to the small temperature difference between the fingers and the environment. Even though the environment temperature was different, the voltage output of the sensors still maintained a gradually increasing trend as the fiber number increased.

The response speed is also an evaluation indicator for the as-synthesized sensor. In Figure 8c, we analyzed the response time of an S1 sensor. When the finger pressed the side of S1 (room temperature of 27 °C), an output voltage of 90 μV was rapidly generated. After removing the fingers, the voltage difference gradually vanished. Figure 3c shows that the time required for the output voltage of S1 to reach 90% of the peak value is 2.7 s. After removing the finger, the output voltage is restored to 10% of the peak voltage for up to 25 s. This may be due to the slow dissipation of the temperature, which remains on the hot side of S1. Under the same temperature difference, the output voltages of the five sensors is significantly different. 

**Figure 9:**
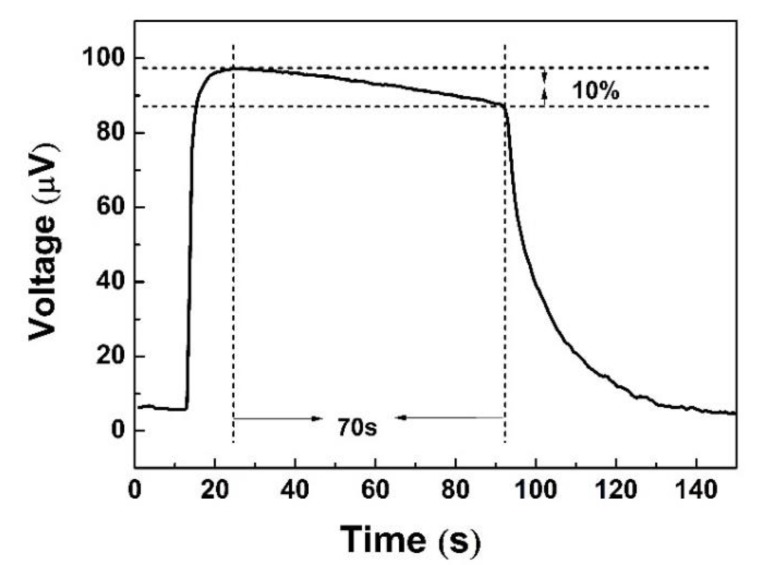
Output voltage curve with the time change for finger touch for 70 s.

**Figure 10:**
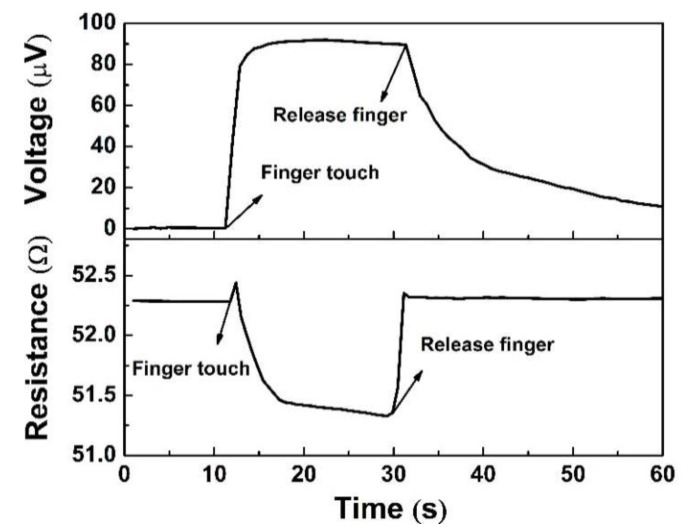
Output voltage and internal resistance of the sensor of S1.

In addition, a test of output voltage for 70 s was applied to analyze the long response time. The output voltage of the device decreased by nearly 10%, and their output voltage curve is presented in Figure 9. At the beginning of the test within 20 s, 98% of the output voltage remained. The major reason can be ascribed to the continually decreasing temperature of the finger, and long-time finger touch is avoided as much as possible. When the sensor was heated up by finger touch, the line had a single primary spike in resistance on the combination of shut decline (Figure 10). This may be caused by fluctuations of the resistance of the contact point. No matter how large the inner resistance changes were, the output voltages only relate to the temperature difference with a regular value of the Seebeck coefficient.

**Figure 11:**
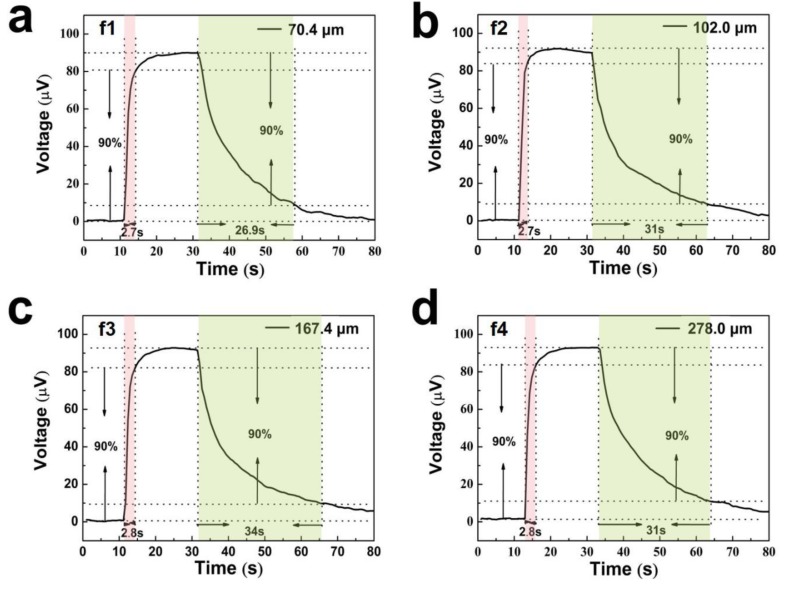
Response time of the single fiber with different fiber diameters: (**a**) f1, (**b**) f2, (**c**) f3, and (**d**) f4.

In our study, no significant difference was observed in the output voltage and response time of fibers with different diameters, which may be due to the small difference in fiber diameters and low response discrimination (Figure 11). However, the voltage recovery time is longer than the heating time, which is related to the heat dissipation speed of the fiber and the temperature of the test environment. If the environmental temperature approaches the finger’s temperature, then the heat dissipation of the sensor may become more difficult. In general, fibers with smaller diameters are more susceptible to being broken than those with larger diameters. Here, the fiber of f1 was used to assemble the sensor.

**Figure 12:**
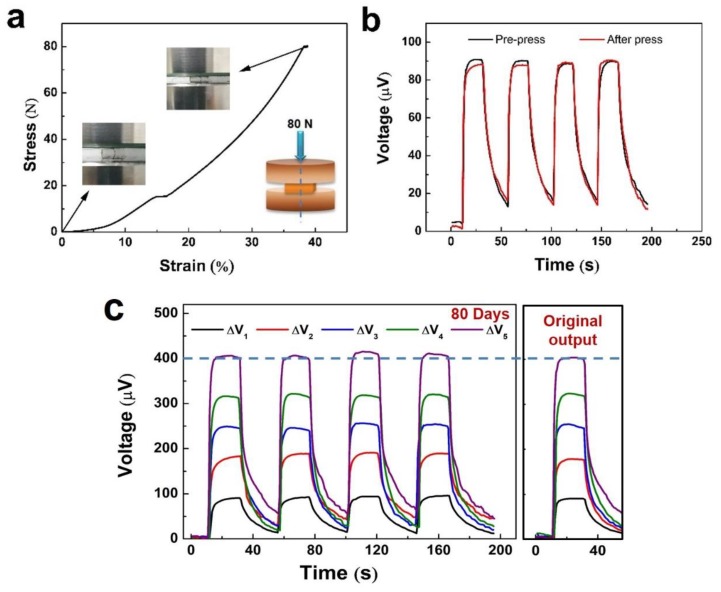
(**a**) Stress–strain curve of a single TE fiber sensor. The inserted picture shows the pressure model diagram during the test. (**b**) Output voltage before and after stress of 80 N. (**c**) Time-variant output voltage curves of the five sensors over 80 days.

The reliability of the sensor depends not only on its performance but also on the influence of external forces on it. To investigate the reliability of the sensor, the stress–strain curve was examined when the device was compressed under fixed stress. The stress–strain curve under the stress of 80 N is shown in Figure 12a. The figure shows the pressure model diagram and the optical image during the test. Obviously, with increasing stress, the thickness of the device was reduced. The original value without stress was 5 mm. Then, it decreased to 3.06 mm under the stress of 80 N. To verify the output voltages of the sensors after the press under 80 N, the responses were also tested again. The output voltage hardly changes compared with the previous test (Figure 12b). However, the sensor can still work without the apparent damage. In general, the stress of the sensor is 15–20 N originating from a human finger touch. In this study, it is not necessary to add much finger force to the sensors, and the output voltage of the sensor only responds with the temperature difference. In addition, the sensors also present a good impact resistance after falling from a height and are not sensitive to ambient humidity. This is attributed to the high flexibility and outstanding stability between PDMS and PEDOT:PSS. It is worth noting that the stress is taken by the sensor rather than by the PEDOT:PSS fiber. The results only show the mechanical property of the sensor when the outside stress is under 80 N. To test the stability of the five sensors, the as-prepared sensors without any encapsulation were placed in air for measuring the output voltages over 80 days. The tests were performed at room temperature (1 atm, relative humidity: 40 ± 3% RH), and the results are shown in Figure 12c. After 80 days, the variations of output voltages of the five sensors are less than 5%, which indicates that the sensors possess a long-time and outstanding stability in air.

### 3.3. Flexible Selector Based on TE Properties

**Figure 13:**
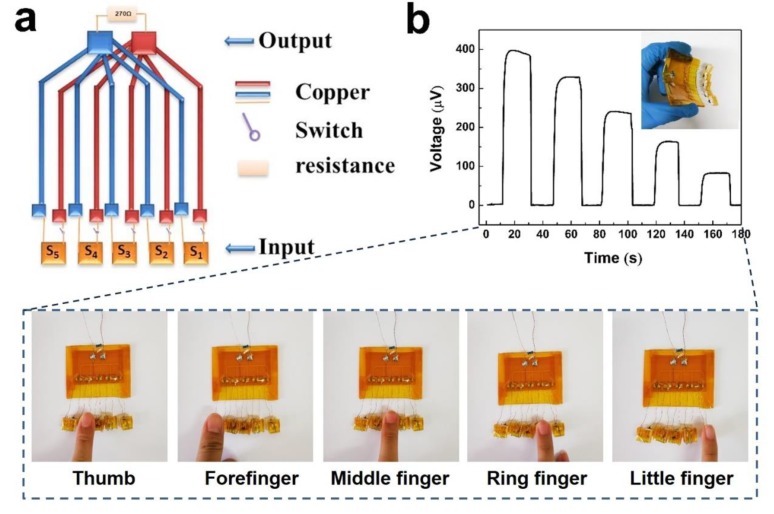
(**a**) Schematic diagram of the selector and (**b**) curve of output voltage of the selector and corresponding optical pictures.

Figure 13a shows a schematic of the selector. To evaluate the performance of the selector, the output voltage of the selector for finger detection is illustrated in Figure 13b. When different fingers touched these sensors, a temperature difference of about 6 °C rapidly developed. Based on the PEDOT:PSS fiber leg number, different output voltages of the selector could be achieved. If one figure touched the sensor, then one signal of voltage could be the output. If two fingers touched the two different sensors, only the lower voltage could be the output. This selector only emitted voltage for a single finger touch, or emitted a lower voltage for two or more fingers touches. Even if the temperatures of the fingers are slightly different, the fluctuation of output voltages is several microvolts. Although the selector platform presents some interesting results, there are still technical challenges in optimizing its circuit, design, and functionality. This work not only provides an initial demo but also reveals its extensive prospects. With a gradual increase of the leg number, the fiber-assembled TE device is highly scalable compared with TE devices assembled in bulk and thin-film form. To connect more PEDOT:PSS fibers in parallel to assemble on the PDMS layer, its high capacity will be expanded.

## 4. Conclusions

In this study, a new structural finger touch sensor is successfully fabricated, which is self-powered, low cost, highly reliable, and reasonably stable. The whole structure of the sensor has a high scalability, and the sensor is simple to prepare. The summit output voltages of the PEDOT:PSS fiber sensors with 1 to 5 legs gradually increase from 90.8 μV to 404 μV when the figures touched the sensors. The temperature difference between the figure and the outside environment is 6 °C. The time for the output voltage to reach 90% of the peak value is only 2.7 s. Despite being exposed to air for 80 days, the output voltages are similar to previous data. In addition, the sensor has a good mechanical property. Under the stress of 80 N for 1 min, the sensors are not damaged, and the output voltages are hardly changed. Given its good stability and pressure resistance, the TE fiber sensor presents a potential application for wearable devices. In recent years, simulated robots have attracted wide attention. If the manipulator has a solid temperature, then our presented method may aid those who wish to use the TE sensor in artificial intelligence. 

## Figures and Tables

**Table 1:** Statistics of TE properties of PEDOT related materials.

Material	Post-treatment reagent	*S* (µV/K)	*σ* (S/cm)	*k* (W/mK)	*ZT*	Type	Reference
PEDOT:PSS	EMIM-DCA	-65	-1500 ~ -1600	——	——	Film	[1]
FS-PEDOT:PSS	——	20.6	2500	0.64	0.05	Film	[7]
PEDOT:PSS/PVA@Ag NPs	——	17	41.5	0.12	3.079 × 10^−3^	Film	[10]
PEDOT:PSS/SWNT	EG	——	450 ± 24	——	——	Fiber	[12]
PEDOT:PSS	EG	14.8	172.5	——	——	Fiber	[13]
PEDOT:PSS/SWNT	NaOH	55.6	1701	0.4 ~ 0.6	0.39	Film	[16]
PEDOT/NWs	H_2_SO_4_ and NaOH	25.5	715.3	——	——	Film	[31]
PEDOT:PSS	EG HCL HCOOH HNO_3_ H_2_SO_4_	——	1128 ± 91 392 ± 29 1289 ± 73 2099 ± 143 2938 ± 325	——	——	Film	[19]
PEDOT:PSS Aerogel	NMP	18.8	35	——	——	Film	[20]
PEDOT:PSS/TeNWs	DMSO	30.8	119	0.168	2.0 × 10^−2^	Film	[20]
PEDOT:PSS	PEG H_2_SO_4_ MeOH DMSO EG	19.95 18.32 18.7 21.76 19.1	882 1851 1202 891 942	——	——	Film	[36]
Hollow PEDOT:PSS	EG	24	——	——	——	Fiber	This study
